# Acute Effects of Instructed and Self-Created Variable Rope Skipping on EEG Brain Activity and Heart Rate Variability

**DOI:** 10.3389/fnbeh.2018.00311

**Published:** 2018-12-11

**Authors:** Alexander John, Wolfgang I. Schöllhorn

**Affiliations:** Institute of Sport Science, Training and Movement Science, University of Mainz, Mainz, Germany

**Keywords:** EEG, learning method, physical activity, acute effects, HRV, recovery, differential learning

## Abstract

The influence of physical activity on brain and heart activity dependent on type and intensity of exercise is meanwhile widely accepted. Mainly cyclic exercises with longer duration formed the basis for showing the influence on either central nervous system or on heart metabolism. Effects of the variability of movement sequences on brain and heart have been studied only sparsely so far. This study investigated effects of three different motor learning approaches combined with a single bout of rope skipping exercises on the spontaneous electroencephalographic (EEG) brain activity, heart rate variability (HRV) and the rate of perceived exertion (RPE). Participants performed repetitive learning (RL) and two extremely variable rope skipping schedules according to the differential learning approach. Thereby one bout was characterized by instructed variable learning (DLi) and the other by self-created variable learning (DLc) in randomized order each on three consecutive days. The results show higher RPE after DLi and DLc than after RL. HRV analysis demonstrates significant changes in pre–post exercise comparison in all training approaches. No statistically significant differences between training schedules were identified. Slightly greater changes in HRV parameters were observed in both DL approaches indicating a higher activation of the sympathetic nervous system. EEG data reveals higher parietal alpha1 and temporal alpha2 power in RL compared to both DL schedules immediately post exercise. During the recovery of up to 30 min, RL shows higher temporal and occipital theta, temporal, parietal and occipital alpha, temporal and occipital beta and frontal beta3 power. In conclusion, already a single bout of 3 min of rope skipping can lead to brain states that are associated with being advantageous for cognitive learning. Combined with additional, cognitively demanding tasks in form of the DL approach, it seems to lead to an overload of the mental capacity, at least on the short term. Further research should fathom the reciprocal influence of cardiac and central-nervous strain in greater detail.

## Introduction

Analyzing effects of physical activity (PA) on cognition or related brain activity has received increasing interest over the past decades (Etnier et al., 1997; Crabbe and Dishman, 2004; Chang et al., 2012b; Cox et al., 2016). Beside aerobic exercise (Tomporowski, 2003; Schneider et al., 2009a,b; Brümmer et al., 2011a; Ludyga et al., 2016a) researchers have been investigating the effects of resistance exercises (Chang et al., 2012a, 2014) and exercises that involve more cognitive demands (Budde et al., 2008; Lambourne and Tomporowski, 2010; Pesce and Audiffren, 2011; Pesce, 2012). Hereby the focus was in majority on the behavioral effects of acute (Tomporowski, 2003; Crabbe and Dishman, 2004; Budde et al., 2008; Schneider et al., 2009a,b; Brümmer et al., 2011a; Pesce and Audiffren, 2011; Chang et al., 2012a, 2014; McMorris et al., 2015), and chronic exercise (Etnier et al., 1997; Cox et al., 2016) dependent on the intensity, duration and type of movement. Hereby effects of acute exercise are understood as the reactions on a single bout of PA. In the chronic case, the effects on repeated, specific PA mostly over a period of more than 1 week or PA already integrated into everyday life are meant. At least the minimum duration of coordinative exercise lasted about 10 min (Budde et al., 2008) and in case of aerobic work the minimum duration was about 20 min (Lambourne and Tomporowski, 2010). Quite recently the studies became more concentrated on the consequences of PA on brain activity.

For scrutinizing the brain activity in connection with whole body movements, EEG is preferably applied. Typically the EEG-signals are decomposed into frequency bands ranging from delta to gamma bands. The delta band (1–4 Hz) oscillations span a rather wide region of neural networks possibly in an inhibitory manner as during sleep (Harmony, 2013). They are also observed during motivational (Knyazev, 2012), attentional (Lakatos et al., 2008; Schroeder and Lakatos, 2009) as well as in concentration processes located in frontal regions (Harmony, 2013). With respect to motor control, delta and theta band activity increases during movement preparation as well as after movement inhibition (Savostyanov et al., 2009). The theta band (4–7.5 Hz) is often associated with the modulation of short-term memory tasks (Klimesch, 1999). The appearance of theta frequencies in the frontal–central areas has been seen when tasks related to working memory processing (Grunwald et al., 1999; Hsieh and Ranganath, 2014) and cognitive control (Cavanagh and Frank, 2014; Cavanagh and Shackman, 2015) were given. Related to motor control, better performance in golf sport and rifle shooting was related to higher fronto-midline theta and higher parietal upper alpha power (Baumeister et al., 2008; Doppelmayr et al., 2008). Increment of theta activity above resting levels was shown at higher workloads during a graded cycling exercise and at exhaustion (Bailey et al., 2008). The alpha frequency band (8–13 Hz) more often correlates positively with the processing speed of information (Klimesch, 1999). More power in the lower alpha band is measured with respect to attentional demands (Klimesch et al., 1993). The upper alpha band is frequently connected with processing of semantic information (Klimesch, 1999). Alpha activity also seems to be increased during short- and long-term memory processes as well as during working memory processes (Basar et al., 1997). However, the effects of PA on the alpha frequency band are widely inconsistent (Cheron et al., 2016). On one side a reduction of alpha power after PA was found (Mechau et al., 1998; Doppelmayr et al., 2008; Schneider et al., 2009b; Ludyga et al., 2016b) and on the other side an increase (Mechau et al., 1998; Bailey et al., 2008; Schneider et al., 2009b; Brümmer et al., 2011b) of alpha power was measured. Beta frequency band (13–30 Hz) seems to play a central role in processing of sensorimotor information (Cheron et al., 2016) and hence to maintain the status quo (Engel and Fries, 2010). Additionally, increasing beta power is involved in conscious thinking as well as in problem solving processes especially in frontal–central areas (Malik and Amin, 2017). With respect to functional separation in sub-bands (Abhang et al., 2016), beta1 activity (12–15 Hz) is seen in connection with focused and introverted concentration. Beta2 activity (15–21 Hz) can be associated with an increment of anxiety and performance. Even higher levels of anxiety, serious stress and high arousal are described in connection to beta3 activity (18–40 Hz) (Abhang et al., 2016). Low beta frequency (13–20 Hz) in the sensorimotor area seems to be connected to sympathetic activity regulating heart rate (Triggiani et al., 2016). Regarding physical exercise, fine motor movement caused a decrease of beta power in primary motor cortex (Kuo et al., 2014). Also preparation and execution of more complex grasping movements were defined by a reduction of beta power in centro-parietal areas (Zaepffel et al., 2013). During steady contractions, beta activity is enhanced in all motor areas (Baker, 2007). Gamma frequency (30–70 Hz) is related to object representation (Herrmann et al., 2004) and is involved in processes related to attention, working and long-term memory (Jensen et al., 2007; Malik and Amin, 2017). Furthermore conscious perception is linked to gamma activity. During the preparation and execution of movements gamma activity is promoted in the motor cortex (Schoffelen et al., 2005). In this study, we focus on the analysis of theta, alpha, beta, and gamma frequency bands.

Because of the sensitivity of EEG-signals on vibrations, a multitude of stimuli and noise during complex whole body movements, EEG in these situations is quite frequently measured immediately after the performance in a quasi-static situation. Instantaneous measurement of post-task effects provides insight into the process of motor consolidation due to existing local phenomena immediately after task execution (Tanaka et al., 2011; Buschkuehl et al., 2012; Crupi et al., 2013). These phenomena seem to be dependent on the type of task and location of the cortex (Landsness et al., 2011; Perfetti et al., 2011). Only for difficult tasks an increase in alpha activity was shown in the cortical area relevant for task execution (Osaka, 1984). Independent of task difficulty, higher alpha and theta power in frontal and posterior regions were found during a fine motor movement like in a sequence learning task. After sequence learning, post-task effects revealed higher alpha power in occipito-parietal areas. Based on these results, the process of learning is seen in a certain correlation with changes in frontal theta and an increment of alpha activity in occipito-parietal areas (Moisello et al., 2013). Transferability of post-task traces in case of motor learning was shown by changes in primary motor and sensory areas after fine motor tasks (Ghilardi et al., 2000).

With respect to the duration of exercises, 21–60 min of running in low intensity with a mean of 39.9, SD 11.4 min and 50–55% of individual aerobic maximum capacity (VO2max) resulted immediately after exercise in short-term increased electroencephalographic (EEG) alpha1 power in parietal and occipital areas. High intensity running with a mean of 38.1, SD 11.1 min and 80–85% of VO2max induced a reduction of EEG alpha1 activity up to 15 min post-exercise (Schneider et al., 2009a). The influence of the familiarity of cyclic exercises on EEG (Schneider et al., 2009b; Brümmer et al., 2011a) was investigated by comparing effects of running, bicycle riding, or arm cranking with moderate (50% VO2max) and high (80% VO2max) intensity for overall 30 min (Brümmer et al., 2011a). After moderate intensity, higher EEG alpha activity in somatosensory was found for familiar movements and after unfamiliar movement types in emotional brain areas. In high intensity exercise only treadmill running, i.e., most familiar mode of movement, was followed by a decrease of beta frequency in the frontal cortex (Brümmer et al., 2011a).

Previous studies mainly focused on the effects of specific movements on brain activation. The influence of movement sequences or variations in movement order on electrical brain activity is widely neglected. Here movement sequences are understood in a way used in different motor learning approaches. Most recently a few fMRI studies on fine motor skills provided a first hunch about the areas of activation dependent on the acquisition schedule of sequential fine motor skills (Lage et al., 2015). A moderating role in the acquisition of movements is also assigned to the colloquial complexity of movements (Tomporowski and Pendleton, 2018). The influence of different motor learning approaches related to the amount of coordinative variability on electrical brain activity has been rarely investigated (Henz et al., 2018). By means of a within-subject design effects of a single, more coordinative demanding 20-min bout, badminton serves (below than 10%-VO2max) according to repetition learning (RL) and differential learning (DL) were investigated (Henz and Schöllhorn, 2016). DL (Schöllhorn, 2000) is based on principles of dynamic systems (Haken, 1970; Glansdorff and Prigogine, 1971; Haken et al., 1985) and neurophysiology (Kandel et al., 1995). DL mainly relies on the fact that only differences allow learning. In contrast to traditional learning, DL considers errors no more as destructive for learning progress but rather as essential fluctuations in living systems that have a constructive influence on learning. Especially, when these fluctuations are increased, the system becomes more instable and less energy is needed for the initiation of self-organized, optimized learning (Schöllhorn et al., 2006, 2009b; Frank et al., 2008). For analyzing the different effects of repetitive and differential schedules, sessions were performed consecutively on 1 day in randomized order and EEG brain activity was measured before and after. The results showed enhanced frontal theta activity and occipito-parietal and central alpha activity following DL compared to RL (Henz and Schöllhorn, 2016). In a follow-up study (Henz et al., 2018) subjects performed two DL realizations (gradual and chaotic), contextual interference learning (CI) (Magill and Hall, 1990; Wright et al., 2016) and RL in the same setup as in Henz and Schöllhorn (2016). All four motor learning approaches were conducted in randomized order on a single day. CI mainly describes an effect (Magill and Hall, 1990; Brady, 2004; Wright et al., 2016) that is observable when more than one fine motor skill is practiced in a random or serial order. During acquisition the performance is interfered, while after a retention period, during actual learning phase, performance is ameliorated. In difference to DL, errors are to be avoided in CI. Therefore, gradual DL was characterized by systematic and mainly expectable variations between subsequent tasks. For example, consecutive task instructions could have been variations in left wrist joint followed by changes in right wrist joint and afterward in right elbow joint. In contrast chaotic DL was based on a random task order with a much smaller predictability for the subsequent movement task (Schöllhorn, 2016). Exemplarily, a chaotic DL schedule could have contained a task sequence of one variation in the left wrist joint, followed by one in the right knee joint, and then by one in the left shoulder joint. Thus a higher degree of unpredictability in chaotic than in gradual DL can be assumed. Similar to the previous study, increased theta and alpha power after both DL methods were identified in somatosensory regions in comparison to RL and CI. Furthermore, chaotic DL resulted in increased theta and alpha activity in motor areas compared to gradual DL and CI. These outcomes were interpreted as an advantageous activation of the somatosensory and motor areas by DL in contrast to RL, probably due to a larger demand of the motor and somatosensory system during DL practice (Henz et al., 2018). From a functional point of view theta and alpha frequency in somatosensory and motor areas are suggested to be positive indicators of motor learning processes (Moisello et al., 2013). In consequence it may be argued motor learning is ameliorated after DL practice. However, all variants of exercises for the DL schedules were instructed and the load on metabolism was low due to the breaks of approximately 10–20 s between the executions of two subsequent variants.

When the effects of learning a bimanual visuo-motor task on brain activity were investigated with regard to the CI paradigm (Pauwels et al., 2018), somehow different results were observed. The study revealed increased brain activity in sensorimotor-related brain regions in blocked compared to random practice. But as a consequence of repeated practice sessions, brain activity of blocked condition decreased and of random practice remained constantly or even increased. After random practice, brain regions related to visual processing were activated in a greater way than after blocked practice (Pauwels et al., 2018).

On a rather phenomenological level several studies have indicated different effects of different motor learning approaches on performance and skill acquisition, especially when RL and DL was compared (Serrien et al., 2018). In experiments on technical skills in team (Schöllhorn et al., 2004; Humpert and Schöllhorn, 2006; Wagner and Müller, 2008; Römer et al., 2009; Hegen and Schöllhorn, 2012) and individual sports (Beckmann and Schöllhorn, 2006; Schöllhorn et al., 2009a) DL resulted in higher acquisition and learning rates than RL. Effects of DL on cyclic movements (Schöllhorn et al., 2009a; Savelsbergh et al., 2010) have rarely been investigated, neither on coordinative aspects nor on metabolism. It remains unclear whether the effects of DL in acyclic exercises will be the same in cyclic exercises with a completely different demand of metabolism in connection with cognitive effort. Additionally, the reasons of evoked effects due to DL are still not clearly explained and therefore the research of DL effects on metabolism and neurophysiological aspects is fostered. Furthermore, due to the methodical standardization of the interventions, the studies on DL were so far characterized by exactly instructed movement executions. DL training with self-created variations as a didactical alternative for causing instability during the acquisition process was not a scientific objective yet. Here instability is understood as a transition between to stable states with increased fluctuations according to dynamic system principle (Schöllhorn, 2000).

The aim of this study was the investigation of the neurophysiological and metabolism effects of different amounts of coordinative variants within a cyclic exercise. Therefore, the electrical brain activity (EEG) and the heart rate variability (HRV) were compared directly before and after rope skipping. Beside the comparison with the effects on RL, one condition of DL exists of receiving instructions about all exercises whereas the other is free in finding variations by themselves. We assumed rope skipping as an exercise with moderate to high intensity dependent of movement duration. Based on the selected study design, rope skipping was expected to be more likely a high intensity activity. We hypothesized, according to the results of a previous study after high intensity running (Schneider et al., 2009a) less reduced alpha1 activity after rope skipping due to the clearly shorter exercise duration. With regard to the comparison of different motor learning approaches (Henz and Schöllhorn, 2016; Henz et al., 2018), we presumed higher theta and alpha activity in somatosensory and motor areas following DL compared to RL after rope skipping. Concerning HRV, we expected a decrease in HRV after DL rope skipping because of predetermined similar heart rates and additional mental load. RPE and especially mental effort were assumed to be higher after both DL sequences as a result of additional mental effort due to various, coordinative task executions.

## Materials and Methods

### Participants

The sample consisted of 10 healthy male volunteers, aged between 19 and 31 with a mean of 24.6, SD 3.31 years to reduce gender and age differences on brain (Wada et al., 1994; Wackermann and Matousek, 1998) and heart activity (Umetani et al., 1998; Kuo et al., 1999). All participants fit the neurologically necessary condition of the same handedness to compare brain activity (Serrien et al., 2006; Sun and Walsh, 2006). Right-handedness was selected as a study participation criterion to facilitate acquisition of possible participants. Volunteers classified themselves as neurologically and cardiologically healthy and mentioned no related medical pre-existing conditions. Physical or cerebral activity influencing substances (Zschocke and Hansen, 2012) have not been consumed at least 24 h before the measurement dates. All subjects confirmed to be able to perform classical rope skipping. Subjects gave their written informed consent for study participation. Participants were coded with numbers for anonymity of personal data. Table 1 gives an overview of the included demographic and lifestyle variables. Ethical standards were complied under the terms of the local institutional ethics committee. The study has been carried out in accordance with the Declaration of Helsinki (2013).

**Table 1:** Demographic and lifestyle variables.

Variables	Type of evaluation
Gender	Male/female
Birth date	Month and year
Neurological impairment	Yes/no
Cardiological impairment	Yes/no
Right-handedness	Yes/no
Ability to perform classical rope skipping	Yes/no
Dependent of experiment day (last 24 h):	
Alcohol consumption	Yes/no, if yes, how much?
Medication	Yes/no, if yes, kind and dosage
Coffee	Yes/no, if yes, how much?


*Variables used to evaluate participants demographic and lifestyle facts.*

### Study Design and Procedure

The study was conducted at the Sports Institute of the Johannes Gutenberg University of Mainz. With a within-subject design the effects of three different coordination related motor learning approaches were investigated. EEG brain activity and electrocardiography (ECG) HRV were chosen as measurement parameters for cognitive and physical performance. Secondary criteria were the subjective state, determined by assessing the Borg scale of perceived exertion (RPE) (Borg, 1982) and mental as well as physical effort, which was rated each on a scale division of integer numbers from 0 to 10. Furthermore, the grade of wellbeing, concentration and sleep were assessed via a classification to good, moderate or bad. Information of the last activity before the test was acquired via individual response of the participants. The measurements were carried out under laboratory conditions. Changes in brightness, volume and temperature were standardized or kept to a minimum.

On three consecutive days one single motor learning approach was conducted in a single bout, subject dependent each day at the same time, 24 h intermission, to reduce time dependent effects (Gundel and Hilbig, 1983; Laitinen et al., 1998). The sequence of motor learning approaches was randomized.

The current subjective state of every participant was identified each session by means of the perceived exertion as well as the grade of wellbeing, concentration, sleep, and the last activity before the test.

**FIGURE 1:**
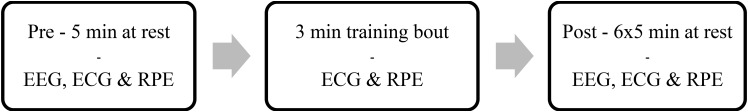
Test procedure.

The procedure of each day (Figure 1) was defined by the measurement of spontaneous EEG activity with eyes open and ECG heart activity for 5 min just before the training bout at rest. The training bout contained 3 min of rope skipping according to one of the motor learning approaches under measurement of ECG activity. This duration was chosen empirically to achieve moderate to high metabolism intensity, which may produce greatest changes in brain activity (Bailey et al., 2008), and to avoid extreme physical exhaustion. Immediately afterward, the recovery process was assessed during 30 min at rest in 5-min intervals, with EEG brain and ECG heart activity measurement. A mean duration of 109, SD 26 s was needed before recovery measurement started. Perceived exertion was rated after each part of the test procedure, particularly every 5 min in post rest measurement. Measurements before and after rope skipping were conducted sitting on an immobile chair with eyes open and watching a smiley picture, fixed on head height in a few meters distance on the wall. Subjects were asked to sit comfortable, but also to minimize their head and eye movements.

### Apparatus

#### Motor Learning Approaches

The three different interventions were defined as the DL with (DLi) and without task instruction (DLc) as well as RL. DL training in general applies movement variations to foster a self-organized learning process by making the system instable by increased fluctuations, which is suggested to help finding an individually optimized solution for a certain physical exercise problem that have to be adapted situationally. Furthermore, no repetition of an ideal, to-be-learned movement execution is recommended and in consequence no error correction has to be given (Schöllhorn, 2000). Regarding consecutive movements in this study the chaotic DL approach was chosen for DLi and DLc to probably achieve greater effects on the brain activity in comparison to gradual DL as had been observed earlier (Henz et al., 2018). To assure high probability of continuous rope skipping without many interruptions, movement variations were conducted with changes only in one movement parameter, i.e., changing only a single joint position or movement. DLi consisted of rope skipping under fast, continuous verbal instructions of different, non-repeated movement tasks (e.g., one-leg jump, rope movement only caused by elbows, rope skipping with head circling or with bent torso) presented by the examiner. The speed of task instructions was set to enable one movement variation per maximally two beats. In case of interruptions of the skipping rhythm, a new task instruction was given for the restart of the movement. Subjects were obliged to perform during DLc as much variations of rope skipping as possible, created by themselves. In both DL approaches repetition of movement variations should be avoided. RL was common, repetitive rope skipping with a frequency of 120 beats per minute (2 Hz) and one feet contact per beat in order to homogenize physical (cardiac) exertion in all motor learning approaches according to exhaustion influence on brain activity and cognitive performance (Coe et al., 2006; Hillman et al., 2008) as well as on the cardiovascular system (Kupari et al., 1993; Dong, 2016). Rope skipping was performed with a steel rope including bearing that was individually adjusted to the anthropometric measures of the subject.

#### Electroencephalography

Spontaneous resting EEG was assessed by means of the EEG-system Micromed SD LTM 32 BS (Venice, Italy) with a sampling rate of 1024 Hz and recorded by the international 10-20 system using 19 electrodes, including Fp1, Fp2, F7, F3, Fz, F4, F8, T3, C3, Cz, C4, T4, T5, P3, Pz, P4, T6, O1, and O2. EEG was recorded before and after training sessions at rest. For all EEG measurements a homogeneous and low impedance (<10 kΩ) of the electrodes in all points was sought. Spectral power was calculated for the theta (4–7.5 Hz), alpha (8–13 Hz), alpha1 (8–10 Hz), alpha2 (10–13 Hz), beta (13–30 Hz), beta1 (13–15 Hz), beta2 (15–21 Hz), beta3 (21–30 Hz), and gamma (30–70 Hz) band. The conduction of brain activity was unipolar with grounding on the nose. Furthermore, a two channel electro-oculogram with electrodes at the medial upper and lateral orbital rim of the right eye was applied. Data were recorded by means of a commercially available software (SystemPlus Evolution – Micromed, Venice, Italy). A first order IIR high pass (0.008 Hz) and a second order IIR low pass (120 Hz) filter was used.

#### Electrocardiography

Electrocardiography was assessed by means of an ECG-recorder (m4medical M-Trace PC) with a sampling rate of 500 Hz and recorded by the international 12-lead derivation. Electrodes of the Einthoven limb leads were placed on both sides of the body one lateral, directly below the clavicle and the other lateral, directly below the costal arch to reduce artifacts during movement. Electrodes of the precordial leads were placed according to the standard location of Wilson et al. (1944). ECG was recorded during the whole test procedure by means of the software Kaunas Load software W04 (Kaunas, Lithuania).

#### Borg Scale of Perceived Exertion and Mental and Physical State

The Borg rating of perceived exertion scale (RPE) was used to evaluate the exertion level right before the training session and directly afterward for a duration of 30 min. During the 30 min every 5 min the exertion level was rated. Subjects read an instruction of RPE 1 day and directly prior to the first measurement to ensure reliable exertion output.

Mental and physical exertion, based on subjective expression of the participants, was documented like the Borg scale right before and after every motor learning approach. This was operationalized using a scale division of integer numbers from 0 for low to 10 for high exertion.

### Data Analysis

Throughout the analysis, a significance level of five percent (*p* < 0.05) was determined. The recorded measurements of brain activity were statistically analyzed by means of MATLAB-based software EEGLAB 14_1_1b (MathWorks, United States; Swartz Center of Computational Neuroscience, San Diego, CA, United States). Data of ECG, specifically HRV were computed by means of MATLAB-based software Kubios HRV 2.2 (Finland) and afterward statistically analyzed by means of the software SPSS 23 (IBM, United States). All variables were tested on standard normal distribution by Shapiro–Wilk test. Depending on the test decision, subsequent tests were calculated either via non-parametric or parametric statistical methods. Descriptive statistics were generated for every sub-region of analysis (Table 2). All other measured data were entered into SPSS.

**Table 2:** Descriptive statistics of selected variables.

		RL	DLc	DLi
Borg scale	Pre	6.6 ± 1.3	6.9 ± 1.9	6.8 ± 1.3
(6 ≤*x* ≤ 20)	Post^∗^	11.6 ± 2.3	14.1 ± 1.8^∗∗^RL	13.1 ± 2.2^∗^RL
	5 min	8.6 ± 2.4	8.8 ± 2.6	9.0 ± 2.2
	30 min	6.8 ± 1.3	6.6 ± 1.3	6.9 ± 1.7
Mental effort	Pre	1.0 ± 0.8	1.5 ± 1.4	1.1 ± 1.1
(0 ≤*x* ≤ 10)	Post^∗^	3.1 ± 1.7	5.3 ± 1.9^∗^RL	5.3 ± 1.9^∗^RL
	5 min	1.7 ± 1.7	1.9 ± 1.4	2.1 ± 1.6
	30 min	1.4 ± 2.8	0.8 ± 0.9	1.6 ± 2.2
Physical effort	Pre	0.4 ± 0.7	0.7 ± 1.3	0.7 ± 0.9
(0 ≤*x* ≤ 10)	Post	5.0 ± 2.1	5.6 ± 1.2	5.4 ± 1.7
	5 min	2.1 ± 2.0	2.3 ± 1.8	2.4 ± 1.8
	30 min	0.5 ± 0.7	0.5 ± 0.8	0.7 ± 1.1
**Heart rate variability**				
Mean heart rate	Pre	66.6 ± 9.7	68.3 ± 10.7	67.3 ± 8.0
(bpm)	Exercise	149.5 ± 15.0	154.6 ± 11.2	155.8 ± 11.3
	5 min	81.4 ± 19.6	85.9 ± 20.5	85.8 ± 17.0
	30 min	81.3 ± 14.4	82.7 ± 12.7	83.1 ± 11.5
Mean RR	Pre	922.9 ± 121.7	905.8 ± 133.9	909.9 ± 101.5
(ms)	5 min	771.5 ± 133.9	741.2 ± 177.0	730.8 ± 140.6
	30 min	760.3 ± 109.2	744.9 ± 106.9	737.2 ± 91.0
SDNN	Pre	59.8 ± 18.7	70.6 ± 20.9	70.6 ± 26.2
(ms)	5 min	51.6 ± 35.0	43.8 ± 34.1	50.4 ± 47.6
	30 min	45.0 ± 23.5	48.1 ± 26.8	44.8 ± 17.3
RMSSD	Pre	61.4 ± 26.3	66.8 ± 26.4	70.0 ± 33.3
(ms)	5 min	56.4 ± 47.5	44.5 ± 43.0	53.4 ± 62.3
	30 min	34.3 ± 21.3	39.3 ± 30.3	33.2 ± 17.9
NN50 (pNN50)	Pre	106.6 ± 61.3 (34.9 ± 23.0)	121.7 ± 59.3 (39.0 ± 21.9)	126.4 ± 54.4 (39.9 ± 20.0)
[# (%)]	5 min	67.4 ± 69.1 (19.4 ± 21.0)	56.8 ± 65.4 (17.2 ± 22.5)	59.2 ± 78.4 (17.2 ± 24.0)
	30 min	43.6 ± 44.7 (12.4 ± 13.6)	54.0 ± 56.6 (15.2 ± 16.5)	44.1 ± 43.0 (11.8 ± 11.5)
LF power	Pre	1720.6 ± 1893.1 (48.8 ± 21.1)	2937.6 ± 2950.2 (50.6 ± 22.9)	3262.1 ± 4528.9 (53.0 ± 19.9)
(ms^2^) (%)	5 min	1611.9 ± 1816.7 (53.2 ± 20.0)	1003.7 ± 1335.3 (52.7 ± 22.1)	1847.4 ± 3003.2 (53.1 ± 19.3)
	30 min	2020.3 ± 1960.4 (71.9 ± 12.3)	1956.0 ± 2303.6 (68.8 ± 21.2)	1574.8 ± 1448.3 (70.8 ± 18.5)
HF power	Pre	1696.9 ± 1328.7 (48.2 ± 22.4)	2324.9 ± 1547.4 (47.0 ± 23.7)	2253.2 ± 1874.4 (45.2 ± 19.8)
(ms^2^) (%)	5 min	1950.0 ± 2672.2 (42.9 ± 20.8)	1374.2 ± 2561.4 (43.4 ± 22.8)	2940.9 ± 5977.9 (42.8 ± 21.4)
	30 min	705.6 ± 853.6 (23.9 ± 12.6)	1066.5 ± 1424.5 (27.6 ± 21.8)	613.9 ± 614.4 (25.8 ± 17.8)
LF/HF ratio	Pre	1.4 ± 1.0	2.1 ± 2.6	2.3 ± 3.7
	5 min	1.9 ± 1.8	2.1 ± 2.2	1.9 ± 1.5
	30 min	4.6 ± 3.9	4.8 ± 3.9	4.6 ± 3.6
Poincaré SD1	Pre	43.5 ± 18.7	47.3 ± 18.7	49.5 ± 23.6
(ms)	5 min	39.9 ± 33.6	31.5 ± 30.5	37.8 ± 44.1
	30 min	24.3 ± 15.1	27.8 ± 21.4	23.5 ± 12.7
Poincaré SD2	Pre^∗^	71.9 ± 21.4	87.1 ± 25.9	85.8 ± 31.8
(ms)	5 min	60.3 ± 37.6	52.6 ± 38.4	59.8 ± 51.8
	30 min	58.7 ± 29.9	61.6 ± 32.4	58.5 ± 21.9
DFA alpha1	Pre	0.9 ± 0.3	1.0 ± 0.3	1.0 ± 0.3
	5 min	1.0 ± 0.3	1.1 ± 0.4	1.1 ± 0.3
	30 min	1.4 ± 0.2	1.3 ± 0.3	1.3 ± 0.3


*Columns defined by motor learning approaches, rows defined by selected variables, mean ± SD, ^∗^ signifies significant intergroup difference, ^∗^RL signifies significant difference to RL motor learning approach, ^∗∗^RL *p* < 0.01.*

#### Psychological State

The output of the variables general well-being, sleep of the previous night and current concentration were transformed into a scale of integer numbers, ranging from 0 for bad, 1 for moderate, to 2 for good. Variables were analyzed by Friedman test differentiating between motor learning approaches. Data of last activity right before each session was scaled in three categories, cognitively and physically demanding as well as without request, and a frequency scale was computed.

#### Electroencephalography

Spectral analysis was used as assessment and interpretation method of EEG data (Zschocke and Hansen, 2012). For each EEG frequency band, theta, alpha, beta and gamma, as well as the respective sub-bands, mean power spectrum of the EEG signal was created by Fast Fourier Transformation with a window size of 1 s and 50% window-overlap. Furthermore, an independent component analysis (ICA) (Makeig et al., 1996) was conducted via EEGLAB. Recurring artifacts, such as eye closing, eye movement, and muscular artifacts were filtered by reducing interference-prone components. After visual inspection of the complete recordings individually occurring, abnormal interferences of the electric potential were eliminated.

For statistical examination, repeated-measure ANOVAs with the within-subject factor defined as motor learning approach (RL, DLi, DLc) were conducted separately for each frequency band and time of measurement (pre rest, 1. post rest 5 min – 6. post rest 5 min). Consecutively to all ANOVAs, *post hoc t*-tests with Bonferroni correction were calculated for pairwise comparison of power spectrum between motor learning approaches. A repeated-measure ANOVA including the within-subject factors as motor learning approach and time of measurement (pre rest, 1. post rest 5 min) was calculated for each frequency band to examine the acute effect of the rope skipping exercise. *Post hoc t*-tests with Bonferroni correction were performed to compare pre rest to post rest power spectrum of each single motor learning approach. For statistical examination analysis of variance included *post hoc* test with Bonferroni correction was conducted. The results were interpreted on the basis of the electrode positions on the scalp according to the Brodmann-areas (Bähr and Frotscher, 2009; Zschocke and Hansen, 2012) and relying on specific functions in connection with the frequency bands.

#### Electrocardiography

Heart rate variability was used as assessment and interpretation method of ECG data. For analysis the second lead (electrode below the right clavicle to electrode below the left costal arch) of the 12-lead derivation was used. Via Kubios HRV, ECG data was inserted and computed for the time intervals of the test procedure. First, correct RR detection was proofed via visual inspection of ECG raw signal and incorrect or missing detection was adjusted. Artifact correction tool was used to eliminate artifacts of RR data. Over all measurements artifact reduction was <5% of total data. Detrending of RR data was conducted applying the method Smooth Priors with λ = 380, resulting in a cut-off frequency of 0.040 Hz (Tarvainen et al., 2002). Frequency analysis was calculated via Fast Fourier Transformation with Welch’s periodogram method and interpolation rate of 4 Hz, window-width of 120 s and window-overlap of 75%.

Time domain (mean RR, mean HR, SDNN, RMSSD, NN50, pNN50), frequency domain of low frequency (LF, 0.04–0.15 Hz) and high frequency (HF, 0.15–0.4 Hz) (power, power %, LF/HF ratio), and non-linear method parameters (Poincaré SD1 and SD2, DFA α_1_) were analyzed (Shaffer and Ginsberg, 2017). Mean RR is defined as the arithmetic mean of the time between two consecutive R-peaks of the ECG signal. SDNN, standard deviation of normal sinus beat intervals, is an indicator of sympathetic (SNS) and parasympathetic nervous system (PNS) activity. RMSSD stands for root mean square of successive differences between normal heart beats and informs about the beat-to-beat variance in HR and is typically taken for the evaluation of the PNS influence on HRV changes. NN50 is a marker of PNS activity and counts the amount of adjacently normal beat-to-beat intervals with a difference of more than 50 ms to each other. pNN50 expresses the percentage of NN50 in comparison to all NN intervals. LF power is defined as the power of HRV-activity with a frequency of 0.04–0.15 Hz and is mainly associated with the activity of the SNS and PNS. HF power reflects primarily the PNS activity. Power % describes the percentage of LF or HF power. LF/HF ratio is the proportion of LF to HF power. Lower ratio indicates PNS dominance, higher ratio relates to SNS dominance. Poincaré SD1 calculates the standard deviation of the ellipse’s width of the Poincaré plot. It is taken as an indicator of short-term HRV and correlates with HF power. Poincaré SD2 measures the standard deviation of the ellipse’s length, provides information about short and long term HRV, and correlates with LF power. DFA α_1_, detrended fluctuation analysis slope α_1_ describes short-term fluctuations via extracting the correlations of adjacent RR intervals over various time scales (Shaffer and Ginsberg, 2017).

Calculated parameters were inserted in SPSS to analyze effects dependent of motor learning approaches. Via repeated-measure ANOVAs with the within-subject factor defined as motor learning approach (RL, DLi, DLc), effects of each standard distributed variable according to all times of measurement (pre rest, post, 1. post rest 5 min – 6. post rest 5 min) were examined. Conforming to non-standard distributed data, relevant variables were analyzed by Friedman test. Consecutively to all ANOVAs, *post hoc t*-tests with Bonferroni correction and subsequently to all Friedman tests, Wilcoxon tests were calculated for pairwise comparison of HRV variables between motor learning approaches. To analyze the acute effect of the rope skipping exercise, a repeated-measure ANOVA containing the within-subject factors as motor learning approach and time of measurement (pre rest, 1. post rest 5 min) was calculated for each variable. *Post hoc t*-tests with Bonferroni correction as well as Wilcoxon tests were performed to compare pre rest to post rest effects of each single motor learning approach.

#### Borg Scale of Perceived Exertion and Mental and Physical State

According to non-standard distributed data, non-parametric Friedman test was conducted to analyze effects between all three motor learning approaches for each measurement. For *post hoc* comparison of two motor learning approaches Wilcoxon test was applied.

## Results

Table 2 displays the descriptive results of selected variables.

### Psychological State

Statistical analysis of questionnaire data yielded a no significant effect of well-being between motor learning approaches [χ^2^(2) = 2.667, *p* = 0.264]. The evaluation of last night’s sleep [χ^2^(2) = 0.500, *p* = 0.779] and the ability to concentrate [χ^2^(2) = 5.200, *p* = 0.074] led also to no significant differences between motor learning approaches. Frequency scale of ‘last activity right before each session’ showed no noteworthy differences between motor learning approaches.

### Electroencephalography

Table 3 displays an overview of significant differences in EEG brain activity between the training approaches and the parameters of statistical analysis.

**Table 3:** Electrodes with significant differences in motor learning approach comparison.

		Electrode	*df*	*F*	*p*	*d*	*Post hoc*	*df*	*t*	*p*	*d*
Theta	Pre										
(4–7.5 Hz)	5 min										
	10 min										
	15 min	O1	2	9.149	0.034	2.016					
	20 min						O1^∗^DLi	9	4.786	0.019	2.14
	25 min	T5	2	9.098	0.035	2.012					
		O1	2	10.055	0.022	2.115
	30 min										
Alpha	Pre										
(8–13 Hz)	5 min						T5^∗^DLi	9	4.907	0.016	2.194
	10 min										
	15 min						P3^∗^DLi	9	4.596	0.025	2.055
	20 min						P3^∗^DLi	9	4.448	0.03	1.989
	25 min	P3^∗∗^	2	12.049	0.009	2.312	P3^∗^DLi	9	4.725	0.021	2.113
	30 min						P3^∗^DLi	9	4.942	0.015	2.21
Alpha1	Pre										
(8–10 Hz)	5 min	P3	2	9.118	0.035	2.012					
	10 min										
	15 min										
	20 min										
	25 min										
	30 min						C3^∗^DLc	9	4.328	0.036	1.936
Alpha2	Pre										
(10–13 Hz)	5 min	T5	2	8.464	0.049	1.941	T5^∗^DLi	9	5.94	0.004	2.656
	10 min										
	15 min						P3^∗^DLi	9	5.017	0.014	2.244
	20 min						P3^∗^DLi	9	4.448	0.03	1.989
	25 min	T5	2	9.337	0.032	2.036	P3^∗^DLi	9	5.65	0.006	2.527
		P3^∗∗^	2	16.868	0.001	2.738
		O1	2	10.983	0.024	2.098
	30 min						T5^∗^DLi	9	4.171	0.046	1.865
Beta	Pre						P3^∗^DLi	9	5.055	0.013	2.261
(13–30 Hz)	5 min										
	10 min										
	15 min										
	20 min						T5^∗^DLc	9	4.313	0.037	1.929
							O1^∗^DLi	9	5.21	0.011	2.33
	25 min	O1	2	10.983	0.015	2.211	O1^∗^DLi	9	4.693	0.02	2.099
	30 min						T5^∗^DLi	9	4.366	0.034	1.953
							O1^∗^DLi	9	4.22	0.043	1.887
Beta1	Pre										
(13–15 Hz)	5 min										
	10 min										
	15 min						P3^∗^DLi	9	4.57	0.026	2.044
	20 min	O1	2	8.75	0.042	1.972	O1^∗^DLi	9	5.731	0.005	2.563
	25 min	T5	2	10.983	0.024	2.098					
		O1	2	10.179	0.021	2.128
	30 min	T5	2	8.656	0.044	1.96	T3^∗^DLi	9	4.518	0.028	2.021
							T5^∗^DLi	9	5.027	0.014	2.248
							P3^∗^DLi	9	4.249	0.041	1.9
Beta2	Pre										
(15–21 Hz)	5 min										
	10 min										
	15 min						P3^∗^DLi	9	4.423	0.032	1.978
	20 min						T5^∗^DLc	9	5.016	0.014	2.243
							O1^∗^DLi		5.475	0.007	2.448
	25 min	O1^∗∗^	2	12.199	0.009	2.33	P3^∗^DLi	9	4.122	0.049	1.843
							O1^∗^DLi	9	4.957	0.015	2.217
	30 min										
Beta3	Pre										
(21–30 Hz)	5 min										
	10 min										
	15 min	F3	2	11.258	0.013	2.238	F3^∗^DLi	9	4.464	0.03	1.966
	20 min										
	25 min	F3	2	8.874	0.04	1.984					
	30 min	F3	2	9.262	0.033	2.028	T5^∗^DLi	9	4.185	0.045	1.872


*Rows defined by frequency band, each divided by time of measurement, ^∗∗^*p* < 0.01, ^∗^DLi signifies significant difference between RL and DLi, ^∗^DLc signifies significant difference between RL and DLc.*

EEG data comparing the three different sessions revealed no significantly increased power in any frequency band before rope skipping at rest.

No significant changes in comparison of power spectrum before and directly after rope skipping were found in any motor learning approach. An illustration of this comparison has been spared out of clarity reason due to the three initial test measurements and their individual differentiation of frequency bands. No significant differences between schedules DLi and DLc in any measurement were identified.

**FIGURE 2:**
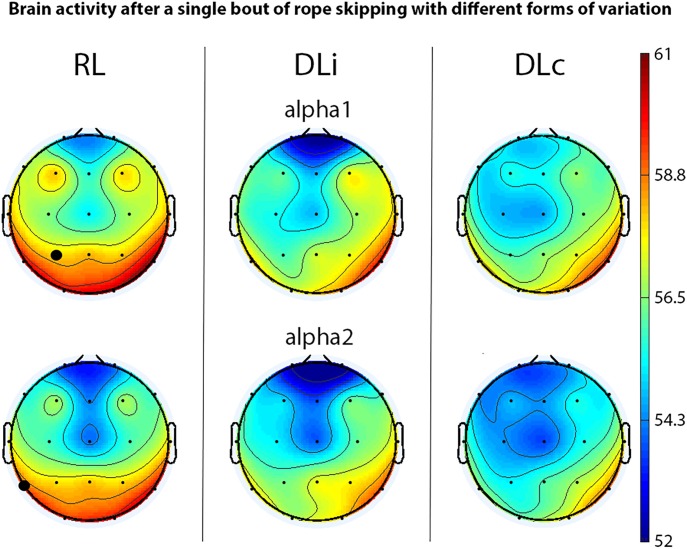
EEG spectral power (only significant effects) of motor learning approaches in first 5 min of recovery after rope skipping. Black bold circles show significant differences (*p* < 0.05) in motor learning approach comparison. Scale unit μV^2^.

After rope skipping, in the first 5 min of recovery alpha1 in electrode P3 and alpha2 power in electrode T5 showed significant effects between all motor learning approaches with higher power in RL (Figure 2). Comparing two training schedules in the first 5 min, just DLi and RL led to a significant difference of alpha and alpha2 power in electrode T5, allocating higher power in RL.

No significant difference was detected in the second 5-min interval.

In the third rest interval (15 min) a significant difference of theta activity in O1 and of beta3 activity in F3 electrode between all training schedules with higher power in RL was found. In comparison of DLi and RL, power of DLi was significant lower in electrodes P3 alpha, alpha2, beta1 and beta2, and F3 beta3 frequency.

Analysis of fourth rest interval (20 min) resulted in an overall approach dependent significant difference in beta1 activity in electrode O1. A significant difference between DLc and RL in beta and beta2 activity in electrode T5, and between DLi and RL in theta, beta, beta1 and beta2 activity in electrode O1 as well as in alpha and alpha2 activity in electrode P3 was observed.

**FIGURE 3:**
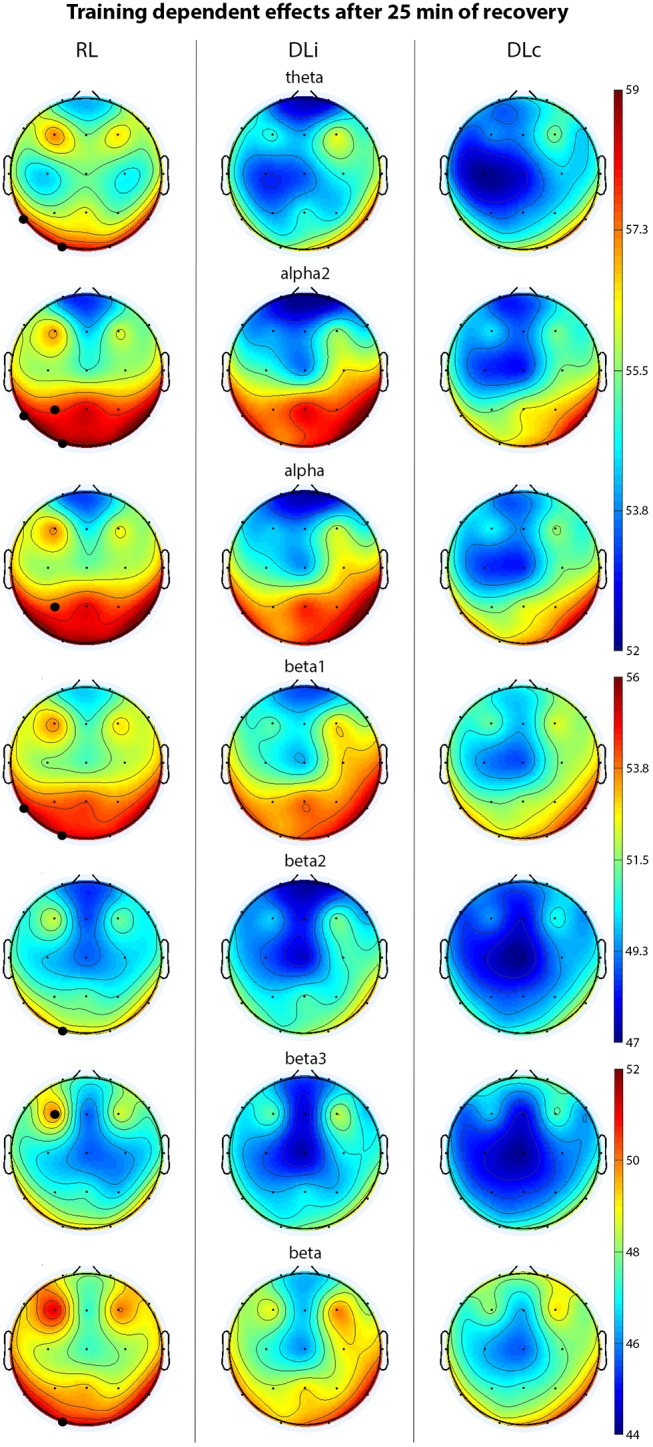
EEG spectral power (only significant effects) of motor learning approaches in penultimate 5 min of recovery. Black bold circles show significant differences (*p* < 0.05) in motor learning approach comparison. Scale unit μV^2^.

Penultimate rest interval (25 min, Figure 3) revealed significant higher power in RL comparing all approaches in theta in electrode T5 and O1, alpha in electrode P3, alpha2 in electrode T5, P3 and O1, beta in electrode O1, beta1 in T5, O1, beta2 in O1, and beta3 in F3 activity. In contrast to DLi, RL approach led to significant higher alpha and alpha2 in electrode P3, beta in O1 and beta2 power in electrode P3 and O1.

In the last 5 min of recovery (30 min), overall higher power was determined in RL in beta1 in electrode T5 and beta3 in F3. Between DLc and RL alpha1 activity in C3 was significant different with higher values in RL. RL showed in contrast to DLi higher power in alpha in P3, alpha2 in T5 and P3, beta in T5 and O1, beta1 in T3, T5 and P3 and beta3 in T5 activity.

### Electrocardiography

Statistical analysis revealed no significant differences in pre rest measurement, except a global effect in Poincaré SD2 [*F*(2) = 3.857, *p* = 0.04, Cohen’s *d* = 1.31] between motor learning approaches. The pairwise comparison of motor learning approaches was not significant.

No significant difference was identified between mean HR of the different training schedules [*F*(2) = 3.308, *p* = 0.06, Cohen’s *d* = 1.21].

During the 30 min recovery measurement no significant effect in any HRV parameter between motor learning approaches could be identified. Regarding analysis of pre to first post rest measurement each motor learning approach showed significant changes. Table 4 displays all significant HRV variable changes dependent on each motor learning approach including the presentation of relevant statistical parameters. RL had a significant difference in meanRR, meanHR, NN50 and pNN50. Both DL approaches showed a clearly higher reduction of NN50 and trivially of pNN50. DLi led to significant changes in the same parameters as RL with additional significance in LF power. HRV of DLc changed significantly like RL, but in addition with effects in SDNN and Poincaré SD2.

**Table 4:** Statistical parameter of significant HRV variables in pre to post exercise comparison.

	RL	DLc	DLi
meanHR	*Z* = -2.803, *p* = 0.005, *d* = 3.829	*T*(9) = -3.988, *p* = 0.003, *d* = -1.783	*T*(9) = -5.243, *p* < 0.001, *d* = -2.345
meanRR	*T*(9) = 7.823, *p* < 0.001, *d* = 3.499	*T*(9) = 4.818, *p* < 0.001, *d* = 2.155	*T*(9) = 7.184, *p* < 0.001, *d* = 3.213
SDNN		*T*(9) = 2.893, *p* = 0.018, *d* = 1.294	
NN50	*Z* = -2.497, *p* = 0.013, *d* = 2.574	*Z* = -2.701, *p* = 0.007, *d* = 3.285	*Z* = -2.599, *p* = 0.009, *d* = 2.885
pNN50	*Z* = -2.803, *p* = 0.005, *d* = 3.829	*Z* = -2.701, *p* = 0.007, *d* = 3.285	*Z* = -2.599, *p* = 0.009, *d* = 2.885
LF power			*Z* = -2.293, *p* = 0.022, *d* = 2.106
Poincaré SD2		*T*(9) = 3.167, *p* = 0.011, *d* = 1.416	


*Columns defined by motor learning approach, rows defined by HRV parameter with a significant difference in comparison of pre to first 5 min of post rest measurement, statistical parameter according to parametric (*t*-test) or non-parametric (Wilcoxon test) analysis.*

### Borg Scale of Perceived Exertion and Mental and Physical State

Comparing the measurement at rest before rope skipping of all sessions no significant difference in perceived exertion [χ^2^(2) = 1.200, *p* = 0.549], in mental [χ^2^(2) = 0.667, *p* = 0.717] and physical effort [χ^2^(2) = 0.875, *p* = 0.646] has been identified. Immediately after motor learning approach execution there was a highly significant difference in perceived exertion [χ^2^(2) = 10.765, *p* = 0.005] and mental effort [χ^2^(2) = 9.135, *p* = 0.01], but not for physical effort [χ^2^(2) = 1.188, *p* = 0.552]. During 30 min of recovery no significant differences in any of the parameters were found.

The comparison of DLc and RL showed a highly significant effect in perceived exertion (*Z* = -2.675, *p* = 0.007, Cohen’s *d* = 3.17) and mental effort (*Z* = -2.388, *p* = 0.017, Cohen’s *d* = 2.30) after acute rope skipping. Similar results after acute training were calculated comparing DLi and RL (perceived exertion *Z* = -1.980, *p* = 0.048; Cohen’s *d* = 1.61, mental effort *Z* = -2.311, *p* = 0.021, Cohen’s *d* = 2.14). Between the differential learning approaches no significant difference over all measurements was determined.

## Discussion

This study aimed to investigate and compare the influence of different, coordination related motor learning approaches combined with a medium physical load on EEG brain activity, metabolism (HRV) and rate of perceived exertion (RPE). In accordance with the current state of research (Schneider et al., 2009a), we hypothesized reduced alpha1 activity after rope skipping, as a rather high intensity activity of short duration. Due to a clearly shorter exercise duration, a less reduction than after running for approximately 30 min. (Schneider et al., 2009a) was expected. Regarding the comparison of different motor learning approaches (Henz and Schöllhorn, 2016; Henz et al., 2018), we expected higher theta and alpha activity in somatosensory and motor areas following DL compared to RL after rope skipping. HRV analysis should reveal a decrease in HRV for the DL conditions because of predetermined similar heart rates and additionally cognitive stress. RPE and mental effort should indicate a higher rating in DL approaches compared to RL due to variously executed, coordinative demanding movement tasks.

Rest measurements prior to rope skipping yielded no significant differences in any of the parameters. Accordingly, a homogenous initial state regarding brain and heart activity as well as perceived exertion for all conditions could be assumed.

In consistence with the hypothesis immediately after rope skipping, RPE was significantly higher for both DL realizations than for RL, no matter whether the variations were instructed or self-created. According to significantly higher mental load and no significant difference in physical exertion, we assume that the effects of RPE are caused by higher demands of mental processes in DL. This is supported by insignificantly varying mean heart rate during rope skipping portending similar metabolism intensity of motor learning approaches. Between DLi and DLc no significant effects in perceived exertion were calculated.

Heart rate variability analysis immediately after strain indicated no significant effect on motor learning approach, but effects when comparing pre to first post measurement in each motor learning approach. Each bout of any motor learning approach resulted in significant changes of HRV-related parameters like meanRR, meanHR and NN50 suggesting cardiovascular strain due to the rope skipping intervention. Interestingly, DLi led to additionally significant lower LF power, DLc lowered SDNN and Poincaré SD2 significantly. Both DL approaches were followed by a clearly higher reduction of NN50 and pNN50. Thus the hypothesis is verified due to difference in HRV between motor learning approaches, portending higher cognitive demands after DL according to a higher reduction of HRV parameters representing SNS and especially PNS involvement. Higher cognitive demand, defined by greater executive task strain and particularly sustained attention, was correlated with less HRV (Luque-Casado et al., 2016). Regarding a comparison to brain activity, sustained attention is described as an executive function linked with the prefrontal cortex (Alvarez and Emory, 2006). In continuing recovery time (10–30 min) perceived exertion as well as HRV did not differ significantly dependent on motor learning approaches. After 30 min of rest HRV parameters did not return to pre training level indicating no complete cardiovascular recovery.

Contrary to the expected reduction in alpha1-power after short, intensive rope skipping, EEG data revealed no significant intra-conditional effect in any motor learning approach when comparing pre test to first post rest measurements. Due to much longer durations of exercise in various other EEG studies (Schneider et al., 2009a,b; Brümmer et al., 2011a; Henz and Schöllhorn, 2016; Henz et al., 2018) it could be questioned, whether the duration of 3 min for a single bout, in comparison to at least 20 min duration in other studies, was sufficient to expect effects on brain activity related to the different conditions. A recent study without neurophysiological measurements suggesting shorter bouts of aerobic exercise, i.e., three 5-min bouts, to foster cognitive performance, provides evidence for probably likewise positive effects on brain activity (Gejl et al., 2018). Most intriguingly, already in the first 5 min after rope skipping EEG-measurements showed significantly different P3 alpha1 and T5 alpha2 power between motor learning approaches, attributing RL higher EEG-power in comparison with DLi and DLc, contradictory to the expectation. Compared to RL a less relaxed state with reduced electrical activity in somatosensory areas of the lower body, and in consequence less somatosensory processing after DLc and especially DLi is speculated. With respect to lower alpha1 power, both DL realizations may be interpreted as necessitating more attentional demands, particularly in areas representing somatosensory processing (Klimesch et al., 1993). Reduced processing speed of information (Klimesch, 1999) and reduced working memory as well as short- and long-term memory processes (Basar et al., 1997) may be attributed to DL realizations of rope skipping. The higher RPE and especially the reported mental exertion after rope skipping in both DL approaches compared to RL point to the same direction. Similar results revealed the study of Pauwels et al. (2018) that showed less activation in sensorimotor areas caused by random contextual interference schedule compared to repetitive (blocked) training. With regard to the influence of preferring and being familiar with an exercise mode on brain activity (Schneider et al., 2009b; Brümmer et al., 2011a), here the RL approach on rope skipping can be considered as the most familiar exercise as well as the most familiar motor learning approach. Hence increased alpha activity in somatosensory areas after RL in some way confirms the findings of brain activity related effects of moderate-intense familiar exercise modes (Brümmer et al., 2011a). However, in comparison to the interventions that were applied in Brümmer et al. (2011a), the present rope skipping exercise was considerably shorter in duration and lead to comparable brain activation. Due to the increased vertical deflection of the body in general and especially the head during rope skipping in comparison to endurance running additional physiological mechanisms and their interaction with neuromuscular activities should be considered. Beside the rhythmic activation of the leg and trunk muscles during ground contact in combination with the heart and breathing rhythm, also the cyclic changes of the blood pressure and in consequence the rhythmic activation of the vagal system can be assumed as influential parameters. However, only an indirect influence of the 2 Hz skipping frequency is assumed because of two reasons. Firstly, the effect is not observed in both DL conditions despite the same skipping frequency, and secondly, the frequency of 2 Hz corresponds to a frequency band that differs from the identified changes in the EEG alpha (8–13 Hz) and theta (4–7.5 Hz) band. Whether the increased and specific mechanical vibrations of tissues and liquids, caused by rhythmic rope skipping, have a retroactive influence on the neural signal transmission demands for further research. Due to the jumping frequency of approximately 2 Hz during rope skipping a modulation of human microvibration (Rohracher, 1962) could occur that may result in an influence on brain activity. Future investigation should evaluate the effect of different evoked mechanical vibration rates on brain activity. In this context the assumed familiarity of the movement as a cause for the observed EEG phenomena in Brümmer et al. (2011a) could also have been caused by means of the changed vertical deflections and stride frequency that resulted from running with 80–85% intensity.

In comparison to Henz et al. (2018), who identified higher alpha power in somatosensory regions in the DL group after a single bout of a coordinative more demanding learning task, our EEG results differ for the DL groups. In this case a one by one comparison suffers from different objectives and in consequence from different study designs. During the badminton serve task the subjects in Henz et al. (2018) had a substantially lower metabolism strain due to at least 10 s break between two subsequent movements preparing the execution of the coordinative instruction. Furthermore, not only one movement parameter was changed by each movement execution but up to three without any time pressure. The observed increase of theta frequency power in the frontal area after a DL (Henz et al., 2018) schedule have led to the speculation that the larger amount of combinations within a shorter time provoked a kind of overloading in the control instance that was followed by a qualitative mental change in terms of switching off the control. In comparison, during the present study subjects in the DL schedules had much higher time pressure due to a much tighter rhythm with additional coordinative stress either instructed externally or created by themselves. This combination of physical and mental strain seems to hinder switching off the frontal control area and in consequence the other effects seem to be afflicted as well. This speculation is also supported by a study determining an inhibition of self-related brain regions, i.e., medial prefrontal and medial posterior parietal cortex, during a sensorimotor processing task with 1 Hz stimuli rate compared to rest condition and slower rate of stimuli (0.3 Hz) (Goldberg et al., 2006). An even higher rate of stimuli of 2 Hz like in the present study could have led to a further inhibition of these brain regions.

Analysis of the second rest interval (10 min) after rope skipping showed no significant effects on EEG measurements between motor learning approaches as well as in comparison to pre rest measurement. This could be related to the duration of possible short-term effect detection. Bailey et al. (2008) described a return of brain activity to pre-exercise levels after 10 min recovery, but subjects performed a graded exercise test until volitional fatigue on a recumbent cycle ergometer. Because of a different training duration and intensity as well as due to an assumed higher fatigue level at the end, a comparison with this study should be handled with care.

Subsequent recovery time (15–30 min) revealed significant differences in O1 theta and beta, P3 alpha and T5 alpha2 and beta1 as well as F3 beta3 power with highest power in RL. Like in the first post rest measurement reduced electrical activation after DL compared to RL is speculated in sensory areas that are associated with the lower body. The effects and interpretations of the first 5 min of post measurement seem to persist throughout the 30 min of recovery. Reduced beta1 activity could be indicated to minor focused concentration in sensory perception processes after DL compared to RL (Abhang et al., 2016). Furthermore, less activation in visual areas could be attributed to DL training. Thus processing of sensorimotor information in areas corresponding visual functions seems to be reduced (Cheron et al., 2016). Less low-beta frequency may indicate higher sympathetic activity of both DL realizations during recovery (Triggiani et al., 2016) strengthening the interpretation of higher cognitive demands during and after DL based on the HRV results. Concerning higher beta3 power of F3, lower stress and arousal (Abhang et al., 2016) in areas that are associated with impulsion, logical thinking and reasoning in DL versus RL could be speculated. In this context the implemented type of rest measurement during recovery might have been critical. Sitting on a chair and focusing a symbol on the wall with eyes open may induce a tiresome state over time. After training without noteworthy mental strain, like in RL, higher visual processing as well as stress in impulsion and reasoning may evolve as a counteraction to falling asleep. Marginal mental strain in RL is supported by the results of perceived (mental) exertion. In general, we speculate that the reduced activation in somatosensory areas can be considered as a consequence of exceeding the capacity of working memory due to sensorimotor and cognitive demand under time pressure in the DL setup. Whether the excess has been provoked by continuously created tasks, by being confronted with new tasks under time pressure, or by an ongoing evaluation of the executed movement and the individual coping of errors, requires further research. Parallels with studies that revealed interfered performance directly after schedules with high (random) CI in comparison to low (blocked) CI (Shea and Morgan, 1979) combined with the found reduced EEG activity immediately after high CI (Budde et al., 2008; Henz and Schöllhorn, 2016; Henz et al., 2018), lead to the speculation of the same underlying mechanism for both phenomena. Because of a repeatedly observed relaxation from this interfered performance in retention tests of CI studies, it would be of interest, whether same effects could be observed in a follow up test on the subsequent day after DL. Similar to the effects in CI studies, a reduced brain activity after a single bout of DL training could change completely after sleep for a benefit on the following practice day or retention test. This suggestion is supported by a CI study showing increased off-line learning after random compared to blocked training (Wymbs and Grafton, 2009). But subjects were given unlimited amount of time to prepare task responses. However, effects of a single bout of rope skipping are hardly to be compared with a series of rope skipping sessions.

A first coarse insight into the dependency of the character of cognitive strain on self-created (DLc) exercises or instructed ones (DLi) with additional physiological load was tried to be achieved by means of two versions of DL schedules. Comparing DLi and DLc, no statistically significant effects in any measurement parameter were found. However, DLi is assumed to necessitate more resources due to more significant changes than DLc in comparison to RL. This might be caused by unexpected varying task instructions in DLi. DLc instead is defined as choosing following movement variations and their execution on its own. A larger amount of consecutive chaotic tasks is probably reached in DLi as well. In comparison DLc is likely more restricted with respect to (time-)limited mental task repertoire of the subjects. For a more detailed comparison of DL methods, evaluating the quality and quantity of task execution may help in future research.

At least, according to the original interpretation of Fisher statistics the few significant results provide enough basis to be encouraged to go for more similar studies.

## Data Availability Statement

The raw data supporting the conclusions of this manuscript will be made available by the authors, without undue reservation, to any qualified researcher.

## Author Contributions

Both authors listed have made a substantial, direct and intellectual contribution to the work, and approved it for publication.

## Conflict of Interest Statement

The authors declare that the research was conducted in the absence of any commercial or financial relationships that could be construed as a potential conflict of interest.
